# Apathy, but Not Depression, Reflects Inefficient Cognitive Strategies in Parkinson's Disease

**DOI:** 10.1371/journal.pone.0017846

**Published:** 2011-03-18

**Authors:** Sara Varanese, Bernardo Perfetti, Maria Felice Ghilardi, Alessandro Di Rocco

**Affiliations:** 1 Division of Movement Disorders, Department of Neurology, New York University School of Medicine, New York, New York, United States of America; 2 Department of Neuroscience and Imaging, ITAB -Institute of Advanced Biomedical Technologies, University “G. d'Annunzio,” Chieti-Pescara, Italy; 3 Department of Physiology and Pharmacology, Sophie Davis School of Medicine, City College of New York, New York, New York, United States of America; University of Groningen, Netherlands

## Abstract

**Background:**

The relationship between apathy, depression and cognitive impairment in Parkinson's disease (PD) is still controversial. The objective of this study is to investigate whether apathy and depression are associated with inefficient cognitive strategies in PD.

**Methods:**

In this prospective clinical cohort study conducted in a university-based clinical and research movement disorders center we studied 48 PD patients. Based on clinical evaluation, they were classified in two groups: PD with apathy (PD-A group, n = 23) and PD without apathy (PD-NA group, n = 25). Patients received clinical and neuropsychological evaluations. The clinical evaluation included: Apathy Evaluation Scale-patient version, Hamilton Depression Rating Scale-17 items, the Unified Parkinson's Disease Rating Scale and the Hoehn and Yahr staging system; the neuropsychological evaluation explored speed information processing, attention, working memory, executive function, learning abilities and memory, which included several measures of recall (immediate free, short delay free, long delay free and cued, and total recall).

**Findings:**

PD-A and PD-NA groups did not differ in age, disease duration, treatment, and motor condition, but differed in recall (p<0.001) and executive tasks (p<0.001). Immediate free recall had the highest predictive value for apathy (F =  10.94; p = 0.002). Depression and apathy had a weak correlation (Pearson index = 0.3; p<0.07), with three items of the depression scale correlating with apathy (Pearson index between .3 and.4; p<0.04). The depressed and non-depressed PD patients within the non-apathetic group did not differ.

**Conclusion:**

Apathy, but not depression, is associated with deficit in implementing efficient cognitive strategies. As the implementation of efficient strategies relies on the fronto-striatal circuit, we conclude that apathy, unlike depression, is an early expression of executive impairment in PD.

## Introduction

Apathy is a reduction of spontaneous and goal-directed behaviors, making affected individuals less responsive and less engaged in daily activities [1]. As a syndrome, apathy affects three domains of the human being. In the behavior domain, apathy expresses itself as lack of effort, lack of productivity, and dependency on others for structured activities. The cognitive domain is affected as loss of interest in novel experiences. Apathy, finally, expresses itself in the emotional domain as a lack of response to positive or negative events, and as lack of concern about one's problems.

In Parkinson's disease (PD), apathy has a high prevalence, ranging from 17 to 70% [2]. Although apathy and depression have been clearly dissociated as independent syndromes in PD [3], symptoms of apathy and depression may also overlap [4]. Recognition of apathy in PD patients is difficult and requires a structured interview. Several instruments have been developed and validated to this scope [5].

Detecting apathy in PD patients has important prognostic implications, as apathy is a predictive factor for the development of dementia [6] and is associated with cognitive dysfunction [6]–[11]. The majority of the studies have highlighted the presence of executive impairments in apathetic PD patients. Pluck and Brown [7] reported that PD patients with apathy have also a worse performance in memory tasks [12]. At first glance, the diversity of cognitive impairments makes the association between these deficits and apathy somehow difficult to explain and interpret. However, executive and memory domains might share a common cognitive core accounting for the variability seen in apathetic PD patients.

Here, our hypothesis is that in PD, the impaired implementation of novel cognitive strategies has a pivotal role in the inefficient storing and recalling of new information as well as in abstract reasoning and problem solving. We propose that this altered mechanism is the underpinning of both apathy and cognitive dysfunction in PD. The identification of a common core may help to clarify the nature of apathy in the context of PD. Specifically, the primary aim of the current study is to investigate whether impaired implementation of novel cognitive strategies may account for the neuropsychological deficits observed in patients with PD and apathy. As secondary aim, we intended to disentangle in these patients the independent contribution of apathy and depression to the cognitive functioning. Thus, we compared the cognitive performance of apathetic and non-apathetic patients with PD, weighted on the clinical factors that could potentially bias the neuropsychological outcomes. In addition, in order to understand the impact of depression, the neuropsychological scores of depressed and non-depressed patients within the non-apathetic group were investigated separately.

## Methods

### Patients

Forty-eight patients were recruited prospectively and consecutively from a cohort of patients referred to the study by their clinicians at our Movement Disorders Center. To be included in this study they had to meet the UK brain bank Criteria for PD [13], were not being treated with antidepressants, had not been diagnosed with dementia or had a Mini Mental State Evaluation (MMSE) [14] total score below 25, and had to be fluent in English.

In order to standardize the evaluations to the best possible condition, all patients were evaluated in on state and under their regular anti-parkinsonian treatment. Doses of dopaminergic medication were converted to equivalent L-dopa doses (LED) [15].

### Procedures and materials

All the evaluations conducted in this study were previously approved by the NYU Institution Review Board, and all subjects signed a written consent form before undergoing the assessment.

The severity of disease was rated using the Unified Parkinson's Disease Rating Scale (UPDRS) [16] and the Hoehn and Yahr ranging system [17]. Depressive symptoms were rated using the Hamilton Disease Rating Scale (HAMD-17) [18]. Apathy was investigated using the Apathy Evaluation Scale (AES), patient-rated version [19], and as per current recommendation [5] a score ≥38 was considered positive for apathy.

A comprehensive neuropsychological evaluation was conducted to investigate the following cognitive domains: attention, speed information processing, learning, memory (recall and recognition), working memory, and executive functions. Each domain was investigated through measures extracted from multiple neuropsychological tools.

The spatial [20] and the digit span backwards [21] were used to assess working memory abilities. The digit symbol [21] and the visual scanning test from the Delis-Kaplan Executive Function system (D-KEFS) [22] were used to rate attention. The speed information processing was investigated using the number sequencing and the letter sequencing tasks of D-KEFS [22]; in order to exclude the effect of bradykinesia on the test performance, we used the formula: [(raw score- motor speed score)/motor speed]. In the memory domain, short term memory was investigated using the digit [21] and spatial span forward tests [20]; recall was investigated using the immediate free recall, the short delay free recall, the long delay free and long delayed cued scores of the California Verbal Learning Test –II (CVLT-II) [23]; recognition was measured using the delayed recognition score of the CVLT-II [23]; learning was assessed using the total learning slope of the CVLT-II [23]. Executive functions were evaluated using the Wisconsin Card Sorting Test (WCST) - 64 cards version [24]; specifically, 4 measures were extracted: perseverative responses, in order to evaluate the ability to shift; total correct responses, non-perseverative responses and categories completed to evaluate the abstract reasoning. Along with the WCST, the number-letter switching task from the D-KEFS was used to assess the executive control and shifting ability.

A trained investigator conducted all the evaluations in a comfortable room, suitable for motor and neuropsychological testing. Both the clinical and the neuropsychological evaluation required about 1 hour each to be completed and they were conducted in the same day.

For the statistical analysis only raw scores were considered.

### Statistical analysis

The first step of the analysis aimed at testing the cognitive differences between apathetic (PD-A) and non-apathetic (PD-NA) patients. The AES cut-off score of 38 divided the patients into two subgroups: PD-A group included individuals scoring 38 or above, and PD-NA group consisted of subjects scoring below 38.

Differences in gender, Hoehn and Yahr stage and disease side of onset were explored using the χ^2^ test for categorical variables. Differences in age, years of education, UPDRS motor score, HAMD-17 score and LED were investigated using the independent sample t-test. The cognitive profile of the two groups was then compared. For each neuropsychological raw score, we performed univariate analysis of variance, with age, years of education, disease duration and treatment (LED) as covariates (ANCOVA), as these variables might affect cognition. In our model the fixed factor was represented by the group membership (PD-A or PD-NA). In order to examine the relationship between apathy and the specific motor signs of PD, five domains were extracted from the UPDRS III: hypomimia (item 19), tremor (items 20 and 21); rigidity (item 22), bradykinesia (items 23, 24, 25, 26, 31), and axial impairment (item 27, 28, 29, 30) [25]. The group differences were studied with ANCOVA as described above. All the assumptions for using the ANCOVA methods were fulfilled (reliability of covariates, correlations among covariates, and linear relationship between dependent variable and covariate, and homogeneity of variance as revealed by the Levene's test and the variance ratio, also know as Hartley's F_max_). Differences were considered significant when the p values were below 0.05. False Discovery Rate procedure was used to correct for multiple comparisons. After testing the differences in cognition between the groups, to ascertain which variables best predicted apathy scores, all the variables showing significant differences between the two groups in the ANCOVA analyses were entered in a stepwise regression procedure.

A second step of the analysis sought to evaluate the effect of depression on cognitive performance: first, we assessed the relationship between apathy and depression by correlating the AES and HAMD-17 total scores (Pearson index); then, we investigated the overlap of apathy into the HAMD-17 questionnaire by computing Pearson's coefficients between the single HAMD-17 item scores and AES total score. Moreover, applying to the accepted HAMD-17 cut off score of 9 for PD [26], we identified within the NA-PD group, a subgroup without depression (NA-PD non-depressed group) and a subgroup with depression (NA-PD depressed group). The neuropsychological scores of the two subgroups were compared using ANCOVA as described above.

To further ascertain the contribution of depression on the relation between apathy and cognitive functioning we performed multiple regressions procedures (enter method) on those test scores that significantly differed between the PD-A and PD-NA groups. The variables entered into the analysis were: AES score, HAMD-17, age, education, disease duration and LED. The analysis was performed on the entire sample of patients.

All the analyses were conducted using the statistical software SPSS v.17.

## Results

### Demographic and clinical characteristics

Based on the AES cut off score of 38, 23 patients were classified in the PD-A group and 25 were classified in the PD-NA group. No differences were found between the two groups with respect to age, gender, disease severity, disease duration, side of onset, although the HAMD-17 scores were significantly higher in the PD-A group (p = 0.007); apathy was not associated with any specific motor sign and the groups were receiving similar doses of dopaminergic treatments (Table 1).

**Table 1:** Demographics.

	PD-A	PD-NA	Sig.(p)
N	23	25	
age	67.4 (9.2)	67.1 (12.5)	0.9
gender (F/M)	11/8	14/15	0.6
education	14.1 (3.6)	15.3 (3.1)	0.2
disease duration	5.9 (3.6)	8.6 (7.9)	0.1
MMSE	28 (1.8)	29.4 (1.1)	
AES	47.3 (5.5)	29.1 (5.3)	0.000*
HAMD-17	17.6 (6.8)	12 (6.8)	0.007*
UPDRS III	24 (10)	22.6 (10)	0.6
hypomimia	1.5 (0.7)	1.4 (0.6)	0.6
tremor	2.9 (3.5)	2.2 (2.8)	0.4
rigidity	4.8 (3.1)	3.9 (2.7)	0.2
bradykinesia	10.8(4.2)	10.3 (5.3)	0.7
axial impairment	3.8 (1.9)	2.9 (2.2)	0.2
H&Y stage			0.8
stage 1	1	3	
stage 2	13	14	
stage 3	7	7	
stage 4	1	1	
side of onset (R/L)	16/6	12/12	0.8
LED	638 (326.3)	896.8 (594.4)	0.07

All values represent mean (SD). P values have been calculated using independent sample t-test for parametric variables and χ^2^ for categorical variables. P<0.05, FDR corrected.

MMSE =  Mini Mental State; AES =  Apathy Evaluation Scale-patient rated; UPDRS III =  Unified Parkinson's Disease Rating Scale, part III (motor); H&Y stage =  Hoehn and Yahr stage; LED =  L-Dopa Equivalent Dose.

### Neuropsychological performance

PD-A patients performed worse than PD-NA patients in 10 out of the 20 neuropsychological measures.

In the working memory, PD-A groups had lower scores at the backward version of the digit span (p = 0.01). All the other differences were found in the CVLT-II and the WCST-64. Specifically, the PD-A patients had lower scores in the recall after the four trials at the CVLT-II (p = 0.002), in the short delay free recall (p = 0.003), in the long delay free recall (p<0.0001), and in the long delay cued recall (p = 0.001). Delayed recognition was impaired as well in the PD-A patients (p = 0.008), while no difference was found in learning slope (p = 0.6). The WCST-64 revealed that the PD-A group had poor ability in abstract reasoning but not in adaptation to external feedback, with a lower number of correct responses (p = 0.002), higher rates of errors (p = 0.001), and lower number of categories completed (p = 0.001). Notably, the number of perseverative responses did not differ among the groups (p = 0.4). The performance at the D-KEFS was similar in the groups, suggesting that visual information processing and set-shifting are not related to apathy.

The neuropsychological performances of the groups are summarized in Table 2.

**Table 2:** Neuropsychological performance.

Domain	Test	Measure	PD-A	PD-NA	Sig.	η^2^
Short term memory	SS	forward	6.9 (1.6)	7.8 (2.2)	0.15	0.05
	DS	forward	8.9 (2.3)	10.1 (2.9)	0.36	0.02
Recall	CVLT-II	trials 1–4 total	22.8 (5.8)	28 (3.7)	0.002*	0.22
	CVLT-II	short delay free	5.9 (2.3)	7.4 (1.2)	0.003*	0.2
	CVLT-II	long delay free	5 (2.2)	7.2 (1.5)	0.000*	0.31
	CVLT-II	long delay cued	5.1 (2.1)	7.4 (1.9)	0.001*	0.26
Recognition	CVLT-II	delayed recognition	8 (0.8)	8.6 (0.6)	0.008*	0.16
Learning	CVLT-II	trial 4-trial1	3 (1.2)	2.8 (1.1)	0.6	0
Working Memory	SS	backward	5.9 (2.1)	6.7 (2.5)	0.1	0.07
	DS	backward	5.6 (1.9)	7.4 (2.3)	0.01*	0.13
Attention	D-KEFS	visual scanning	35.9 (17.4)	33.2 (12.4)	0.89	0
	DSy		44.9 (16.5)	51.4 (15.2)	0.29	0.03
Speed information processing	D-KEFS	number sequence (weight)	0.4 (0.9)	0.3 (0.5)	0.6	0.07
		letter sequence (weight)	0.6 (1)	0.2 (0.4)	0.2	0.05
		motor speed	52.4 (32)	45 (22.5)	0.3	0.03
Executive functions	WCST-64	total correct	34.9 (10.8)	45.6 (11.2)	0.002*	0.22
		perseverative responses	14.9 (7.9)	12.1 (11.5)	0.36	0.02
		non-perseverative errors	15.6 (8.5)	8.3 (5.2)	0.001*	0.23
		categories completed	1.6 (1.3)	3.2 (1.7)	0.001*	0.23
	D-KEFS	number-letter (weight)	2.5 (1.5)	1.7 (1)	0.07	0.07

All values represent mean (SD). Between-groups comparisons have been investigating using univariate analysis of variance for each variable, with age, disease duration and Led as covariates and group membership (apathy vs. No apathy) as fixed factor (ANCOVA). The η^2^ statistic was used to estimate the effect size. P <0.05, FDR corrected. D-KEFS = Delis-Kaplan Executive Function system; CVLT-II = California Verbal Learning Test –II; WCST-64 =  Wisconsin Card Sorting Test-64 cards version; SS = Spatial Span; DS =  Digit Span; DSy =  Digit symbol.

### Regression analysis

The CVLT-II total recall score proved to be the best predictor of apathy (R = 0.44; F =  10.94; p = 0.002) in the stepwise regression analysis of the neuropsychological measures tested.

### Secondary analysis on depression

Since the PD-A and PD-NA groups showed significant differences in HAMD-17 total score, we conducted a series of analysis to determine the impact of depression on cognitive performance. First, we found a weak association between apathy (AES total scores) and depression (HAMD-17) in the entire PD population (r =  0.30, p  =  0.03). However, since such correlation and the differences between the PD-A and PD-NA groups in the depression scale could have resulted from specific HAMD-17 items reflecting apathy trait, we correlated each HAMD-17 item with the AES total score. Indeed, we found that three out of the 17 items showed positive correlation: item 7, investigating interest in daily work and other activities (Pearson index = 0.33; p = 0.022); item 8, investigating retardation in response (Pearson index = 0.38; p = 0.007); item 13, investigating the somatic general symptoms (Pearson index =  0.38; p = 0.003).

**Figure 1:**
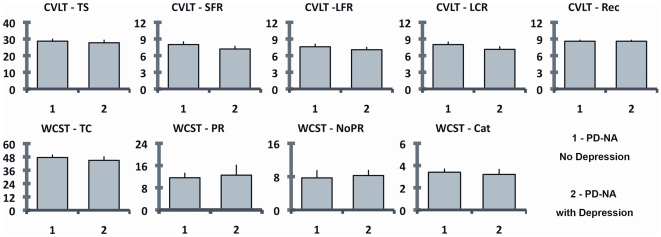
Recall and executive profiles of non-apathetic PD patients with and without depression. The scores are expressed as mean score (the bar shows the standard error). None of the comparisons reaches the statistical significance. CVLT-TS =  California Verbal Learning Test II-Total recall score; CVLT-SFR: California Verbal Learning Test II-Short free recall; CVLT-LFR =  California Verbal Learning Test II-Long free recall; CVLT-LCR =  California Verbal Learning Test II-Long cued recall; CVLT-Rec = California Verbal Learning Test II-Recognition; WCST-TC =  Wisconsin Card Sorting Test- Total correct; WCST-PR =  Wisconsin Card Sorting Test-Perseverative responses; WCST-NoPR = Wisconsin Card Sorting Test- Non-perseverative responses; WCST-Cat =  Wisconsin Card Sorting Test- Categories completed.

In a second step of the analysis, we studied the impact of depression on cognition within the PD-NA sample. Based upon the suggested score for depression in PD [16], fifteen patients with a HAMD-17 score above 9 were considered depressed (Table 3). The scores of the neuropsychological tests of PD-NA patients with and without depression did not differ statistically (Figure 1, Table S1).

**Table 3:** Demographics of non apathetic groups.

	PD-NA	PD-NA	Sig.(p)
	Not depressed	depressed	
N	10	15	
age	62.3 (11.4)	70.33 (12.5)	0.1
gender (F/M)	5/5	6/9	0.9
education	16.3 (0.67)	14.67 (3.8)	0.13
disease duration (years)	7.2 (5.6)	9.4 (9.3)	0.5
MMSE	29.3 (1.3)	29.5 (0.9)	
AES	29.9 (4.9)	28.8 (5.8)	0.7
HAMD-17	5.1 (2.7)	16.7 (4.4)	0.000*
UPDRS III	18.5 (9.3)	25.3 (12.55)	0.09
hypomimia	1.6 (0.5)	1.4 (0.5)	1
tremor	1.9 (2.2)	2.4 (3.3)	0.4
rigidity	3 (1.5)	4.7 (3.2)	0.9
bradykinesia	8.2(5.1)	12.1 (4.9)	0.7
axial impairment	2.5 (2)	3.3 (2.4)	0.7
H&Y stage	I-III	II-III	0.8
side of onset (R/L)	5/5	7/8	
LED	785 (612.8)	971.4 (591.1)	0.45

All values represent mean (SD).

P values have been calculated using independent sample t-test for parametric variables and χ^2^ for categorical variables.

MMSE =  Mini Mental State; AES =  Apathy Evaluation Scale-patient rated; UPDRS III =  Unified Parkinson's Disease Rating Scale, part III (motor); H&Y stage =  Hoehn and Yahr stage; LED =  L-Dopa Equivalent Dose.

Furthermore, apathy resulted the best predictor of cognitive performance in the regression analysis conducted on the entire group to ascertain the individual contribution of apathy and depression. Interestingly, the contribution of depression was not statistically significant (Table 4).

**Table 4:** Differential contribution of apathy and depression on cognitive functioning.

Domain	Test	Model summary	predictors	Beta	SE Beta	Stand Beta	p value
Recall	CVLT-II trials 1–4 total	R^2^ = 0.48	*apathy*	− 0.18	0.68	− 3.44	**.014**
		F_(6,39)_ = 6.1*	*depression*	0.03	0.10	.035	.798
	CVLT-II short delay free	R^2^ = 0.51	*apathy*	− 0.05	0.02	− .24	**.043**
		F_(6,39)_ = 6.7*	*depression*	− 0.05	0.04	− .20	.137
	CVLT-II long delay free	R^2^ = 0.50	*apathy*	− 0.07	0.03	− .35	**.01**
		F_(6,39)_ = 6.5*	*depression*	− 0.01	0.04	− .04	.741
	CVLT-II long delay cued	R^2^ = 0.38	*apathy*	− 0.07	0.03	− .31	**.037**
		F_(6,39)_ = 4.1*	*depression*	− 0.05	0.05	− .17	.256
Recognition	CVLT-II delayed recognition	R^2^ = 0.31	*apathy*	− 0.02	0.01	− .33	**.04**
		F_(6,39)_ = 2.9∧	*depression*	0.02	0.02	.22	.172
Working Memory	DS backward	R^2^ = 0.28	*apathy*	− 0.07	0.03	− .31	.057
		F_(6,39)_ = 2.5∧	*depression*	0.01	0.05	.03	.859
Executive functions	WCST-64 total correct	R^2^ = 0.49	*apathy*	− 0.33	0.15	− .28	**.043**
		F_(6,39)_ = 6.1*	*depression*	− 0.4	0.23	− .24	.094
	WCST-64 categories completed	R^2^ = 0.42	*apathy*	− 0.05	0.02	− .29	**.050**
		F_(6,39)_ = 4.5*	*depression*	− 0.05	0.04	− .21	.162

∧ < .05,

*< .005, in bold the significant value of the predictors.

Regression analysis on the test scores that differentiated PD-NA and PD-A. The variables entered into the analysis were: AES score, HAMD-17, age, education, disease duration and LED. The analysis was performed on the entire sample of patients. Model summary reports the R squared and the results of the ANOVA test for the different dependent variables. We report the beta values only for the two predictors of interest (AES score and HAMD-17 score).

## Discussion

The results of our study support previous evidence of the existence of a distinct subgroup of non-demented patients with PD with cognitive impairments associated with clinically relevant levels of apathy. Importantly, the novel finding is that in PD, apathy, but not depression, is associated with specific deficits of recall and executive functions. Indeed cognitive functioning was best predicted by apathy, while depression had no or negligible effect. These data suggest that abnormal performance of apathetic patients with PD likely results from implementing new and efficient cognitive strategies. Such impairment may be at the basis of the poor performance both in finding new categories in the WCST (abstract reasoning) and in the recall and recognition of words that can be acquired through categorization strategies (CVLT-II) in PD patients with apathy.

Abstract reasoning and strategy development are classically associated with frontal lobe functioning [27] and are sensitive to frontal lesions as well as to functional deficits of the fronto-striatal circuit. Apathy is commonly defined as a primary emotional disorder. However, the model of “cognitive inertia” recently proposed [28] considers apathy as a complex behavioral deficit of self-initiation. This usually occurs as a consequence either of dysfunction of the prefrontal cortex, or of diseases of the basal ganglia which disrupt the associative pathways to the prefrontal cortex. WCST is certainly a useful tool to detect frontal lobe dysfunction. In particular, the WCST is sensitive to deficits of a number of executive abilities as shifting of attention between sets, abstract reasoning, and problem solving. In our study, we observed that apathy in PD was associated with poor planning and rule-finding, but not with set-shifting, pointing to a specific impairment of the ability to generate new cognitive strategies.

In addition, apathy was associated with significant recall and recognition deficits in the CVLT-II. Rather than a primary memory disorder, this impairment is likely due to poor strategy implementation at the encoding and the recall stages. Specifically, a deficit of the encoding of new items may account for the abnormal cued recall and recognition; whereas disruption of recall may produce poor performance during the short-delay and long-delay free retrievals.

In fact, in the CVLT-II, the words to be retained can be more efficiently encoded and recalled by using semantic strategies, as also confirmed by recent findings in patients with focal frontal lesions [29]. This hypothesis, which is in agreement with other studies in patients with traumatic brain injury [30], is supported by our results showing that immediate CVLT-II free recall is the best predictor of apathy. In agreement with a previous study [31], it is unlikely that these differences can be explained by disease severity and dopamine depletion, as the two groups did not significantly differ regarding stage of disease, motor disability, and LED.

The main limitation of this study is that it is difficult to dismiss completely the role of depression in our results, since concomitant depression was not considered as an exclusion criterion. However, the lack of correlation between depression and cognitive functions makes depression alone an unlikely explanation for the difference between apathetic and non-apathetic PD patients. This conclusion is also supported by the results of the regression analysis controlling for depression that demonstrated that apathy was the best predictor of cognitive performance. It might be argued that the apathetic patients had greater depressive scores. However, the weak correlation between apathy and depression was mostly due to the fact that the HAMD-17 contains items that specifically investigate and rate apathetic features. Altogether, our findings are consistent with previous observations that apathy and depression can occur in PD as independent clinical phenomena [3], [31], [32]. The dissociation between apathy and depression has important prognostic and therapeutic implications. Unlike depression, indeed, there is no specific treatment for apathy, although apathy leads the patients to physical inactivity increasing the risk of further functional decline and disability [33]. In addition, identifying apathy with depression may be one of the reasons for the poor response to anti-depressive treatment commonly seen in PD. Indeed, the use of the HAMD-17, which is one of the recommended [26] questionnaire to screen depression and monitor treatment responses in PD, includes items that are apathy-related and that can bring to an incorrect diagnosis of depression.

In summary, we conclude that apathy should be considered an early manifestation of dysexecutive syndrome in PD that reflects a disruption of cognitive processing. Given that apathy is a predictive factor for dementia [6], our findings may serve to encourage the clinicians to conduct extensive neuropsychological investigations in patients showing apathetic symptoms, in order to detect subtle cognitive impairments. Future longitudinal studies will have to ascertain whether apathetic PD patients are destined to develop overt dementia, more than patients not experiencing apathy.

## Supporting Information

Table S1 Neuropsychological scores of PD-NA with depression and PD-NA without depression groups. For each neuropsychological variable the table reports the mean, standard deviation, minimum and maximum together with the 95% confidence interval for mean.
(XLS)
